# Clinical and Radiographic Evaluation of Autologous Platelet-Rich Fibrin With or Without Demineralized Bone Matrix in the Treatment of Grade II Furcation Defects

**DOI:** 10.7759/cureus.44394

**Published:** 2023-08-30

**Authors:** Bhavin Patel, Surabhi Joshi, Tanya Nagrani, Gaurav A Girdhar, Heli Patel, Susmita Sinha, Mainul Haque, Santosh Kumar, Md. Ahsanul Haq

**Affiliations:** 1 Periodontology, Karnavati School of Dentistry, Karnavati University, Gandhinagar, IND; 2 Physiology, Khulna City Medical College Hospital, Khulna, BGD; 3 Karnavati Scientific Research Center, Karnavati School of Dentistry, Karnavati University, Gandhinagar, IND; 4 Pharmacology and Therapeutics, National Defence University of Malaysia, Kuala Lumpur, MYS; 5 Biostatistics, Infectious Diseases Division, International Centre for Diarrhoeal Disease Research, Bangladesh (ICDDRB), Dhaka, BGD

**Keywords:** grade ii furcation defects, therapeutic intervention, autologous, clinical and radiographic evaluation, periodontal disease, demineralized bone matrix, platelet-rich fibrin

## Abstract

Introduction

This study aims to differentiate the employment of demineralized bone matrix (DMBM; Osseograft, Advanced Biotech Products (P) Ltd, Chennai, India) and platelet-rich fibrin (PRF) alone to a composite graft consisting of both materials in the surgical actions toward the anomalies of the human periodontal furcation imperfection.

Methods

In a split-mouth study, 30 patients with mandibular molars affected by the furcation were allocated without conscious choice to test (PRF + DMBM, n = 30) or control (PRF, n = 30) categories. At the starting point, three months after surgery, and six months later, the following modifiable factors were evaluated: probing pocket depth (PPD), full-mouth plaque scores, full-mouth gingival scores, radiographic defect depth, relative vertical clinical attachment level (RVCAL), and relative horizontal clinical attachment level (RHCAL).

Results

Results at three and six months demonstrated substantial differences between baseline values for both treatment methods in clinical and X-ray imaging appraisal. Nonetheless, the PRF/DMBM group manifests statistically significantly soaring changes observed in comparison to the PRF group. Overall, the probing depth (PD) in the test site was significantly lower than that in the control site, showing a reduction of 68% (95% CI=41%, 95%, p<0.001).

Conclusion

Clinical indications significantly improved with PRF and DMBM combined instead of PRF alone. On radiographs, the test group also showed higher bone fill.

## Introduction

Periodontal illnesses affect the tissues that support and surround the teeth in their sockets, and they can result in tooth loss if left untreated [1-3]. Effective periodontal therapeutic intervention aims to rebuild the diseased periodontium's functional capacity and structural integrity [4-6]. Regeneration of the damaged attachment system and restoration of pre-disease anatomy are the ultimate goals of periodontal therapy [7, 8]. Numerous reconstructive therapeutic procedures have been explored for treating intra-bony periodontal disorders [8], including bone grafts and their alternatives, directed tissue regeneration, growth factors, enamel matrix derivatives (EMD), tissue engineering, and combination techniques [9-11].

It has been reported that guided tissue regeneration with bone replacement or transplantation may improve clinical outcomes of periodontal intraosseous defects [12, 13]. The physiological traits of bone grafts and bone substitute materials (BSM) are typically described using osteoinduction, osteoconduction, and osteogenesis [14,15]. Common examples of BSM are β-tri-calcium phosphate ceramics, bioactive glasses, polymer-based bone substitutes, Ca2+ phosphate cements, biphasic Ca2+ phosphates, etc. [16,17]. Allogenic, obtained from a different person of the identical species; xenogenic, acquired from another species; and alloplastic, produced artificially, are the three categories often utilized for periodontal disease intervention [18-20].

An autologous biological scaffold platelet-rich fibrin (PRF), the second-generation platelet concentrate, is created for periodontal therapy [21,22]. Multiple researches reported that bone graft combined with platelet-rich plasma (PRP), PRF, EMD, and amnion membrane have adjunctive effects on the therapeutic intervention for intra-osseous periodontal flaws [23-25], and PRF was found to be the most efficient regeneration accompaniment [23,24]. PRF is a fibrin cast or mold in which platelet cytokines, growth factors, and cells are confined or impounded and freed after an accurate time and that can benefit as an ingestible membrane [26].

Osseous grafts are intended to increase the clinical attachment level (CAL) and promote skeletal regeneration [12,27,28]. Despite its drawbacks, one of the most successful osseous transplants is demineralized freeze-dried bone allograft (DFDBA) [29-33], including developing connective tissue attachment and variable defect resolution [34]. According to Ilgenli et al. [35], PRP alone was weighed against DFDBA with PRP, and in another study by Piemontese et al. [36], PRP and DFDBA fusion was equated to the DFDBA and saline combination. In both investigations, using DFDBA and PRP together had more significant results [35,36].

The principal goal of the current study was to evaluate the clinical and radiographic effectiveness of a composite graft composed of demineralized bone matrix (DMBM; Osseograft, Advanced Biotech Products (P) Ltd, Chennai, India) and PRF vs. PRF alone in the surgical repair of anomalies of the human periodontal furcation. This study tried to accomplish the following objectives: the gain in CAL, both vertically and horizontally, reduced probing depth (PD), and radiographically reduced bone defect depth.

## Materials and methods

**Figure 1:**
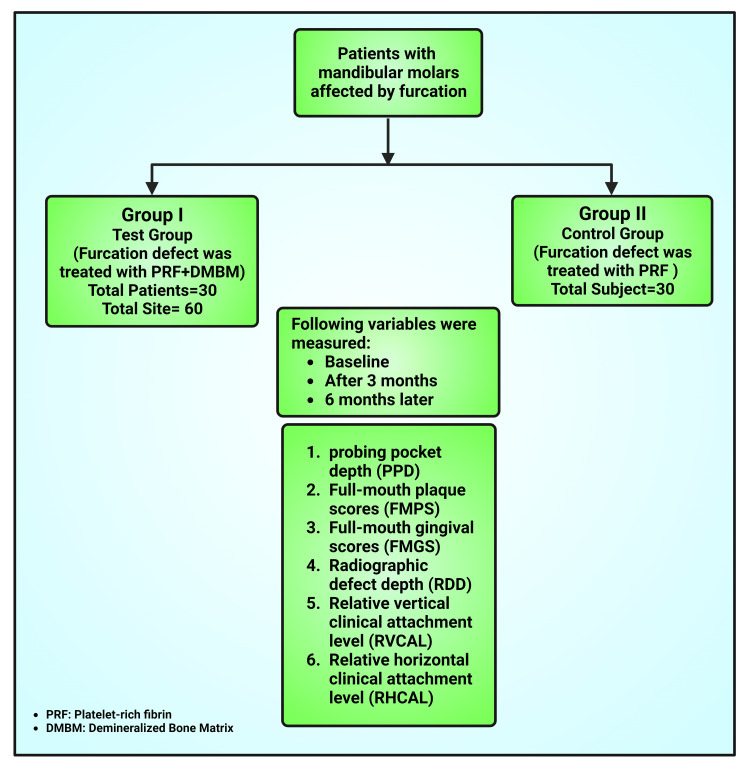
Flowchart showing the methodology of the study Note: This image was created using the premium edition of BioRender (BioRender, Toronto, Ontario, Canada, https://www.biorender.com/) accessed on August 18, 2023, with the license number TY25QQRHCR. Image credit: Susmita Sinha

This was a compare and contrast clinical research evaluating the effectiveness of PRF and DMBM in treating furcation defects to that of PRF alone. The current investigation was organized in the Department of Periodontology at the Karnavati School of Dentistry in Uvarsad, Gujarat, India, for two years from 2013 to 2014. The control group (the total 30 sites) came in for only PRF medication, while the experimental group went through PRF plus DMBM (Figure 1). Clinical outcomes were assessed at three and six months, while patient net results were evaluated throughout the healing process.

Study design and patient criteria

This comparative, single-masked study was conducted, which included 60 furcations involving mandibular molars in 30 patients, and voluntary, informed consent was obtained from all patients. Mandibular molar teeth were selected based on earlier studies and other countries around the globe [37-40]. However, in contrast, furcation-related dental illness involvement is drastically more in the maxillary than in mandibular molars had been reported [41,42]. Each patient was split into two groups in the following ways: Group I (test group): The furcation defect was treated with PRF + DMBM and covered with PRF membrane. Group II (control group): The furcation defect was treated with PRF and covered with PRF membrane. The study comprised 30 individuals with paired contralateral Grade II mandibular furcation abnormalities. Grade II involves bone destruction in one or more parts of the furcation. However, a quantity of the alveolar bone and periodontal ligament endure undamaged. Consequently, it permits mere permeation of the probe into the furcation zone. The radiograph may or may not expose the Grade II furcation association [43,44]. All adult systemically healthy patients were aged 18-65 years. No pediatric population was included in this study.

Inclusion and exclusion criteria

The furcation could only be partially probed for patients who met the inclusion criteria of paired, contralateral Grade II furcation flaws (Glickman's classification). There was no definite probing range. It depends on the individualized level of periodontitis. In general, it was about 5-10 mm. The radiograph may or may not show involvement of Grade II furcation. Pocket PD was ≥5 mm, with endodontically vital, asymptomatic mandibular molars. Those patients who met the mentioned criteria were included in the current study. Patients with systemic illness, those taking medications that possess well-established pharmacology to impede the healing of periodontal wounds, such as Ca2+ channel blockers or corticosteroids, those with drug allergies, lactating or pregnant women, patients who used any sort of tobacco, poor oral hygiene, and teeth with interproximal intra-bony defects, endodontic involvement, or mobility greater than Grade I were excluded.

Methodology of intervention

Researchers first vividly explained the current study's plan, including future scientific publication; additionally, before any surgical or medical interventions were performed, researchers obtained ethical approval and written informed consent from each patient. Scaling and root planing were carried out among all cases of both groups as part of the phase I therapy. All cases of this research received a broad-spectrum antimicrobial that was started the day before surgery and continued up to eight days based on the patient's personalized medical history and need. Depending on the patient's response, a postoperative oral rinse containing 0.12% chlorhexidine gluconate was ordered to be administered twice daily for two to four weeks.

The following clinical data were noted at baseline, followed by postoperative assessments at intervals of three months and six months: plaque index (Turesky-Gilmore-Glickman modification of Quigley Hein) [45], modified gingival index (Lobene 1986) [46,47], PD [48,49], relative vertical clinical attachment level (RVCAL) [50,51], and relative horizontal clinical attachment level (RHCAL) [50,51]. At baseline, three months, and six months, radiographic parameters were noted. Radiographic parameters were recorded with direct digital grid radiographs (radiovisiography) and standard intraoral grid at three and six months at baseline. The distance was recorded between the fornix furcation to the defect base.

Surgical procedure

Intraoral antisepsis was the first step; mucoperiosteal flaps were reflected after local anesthetic injection; buccal and lingual sulcular surgical slit were performed; meticulous defect debridement and root planning were carried out with an ultrasonic tool and 5-18 Gracey curettes were utilized; no osseous recontouring was performed.

**Figure 2:**
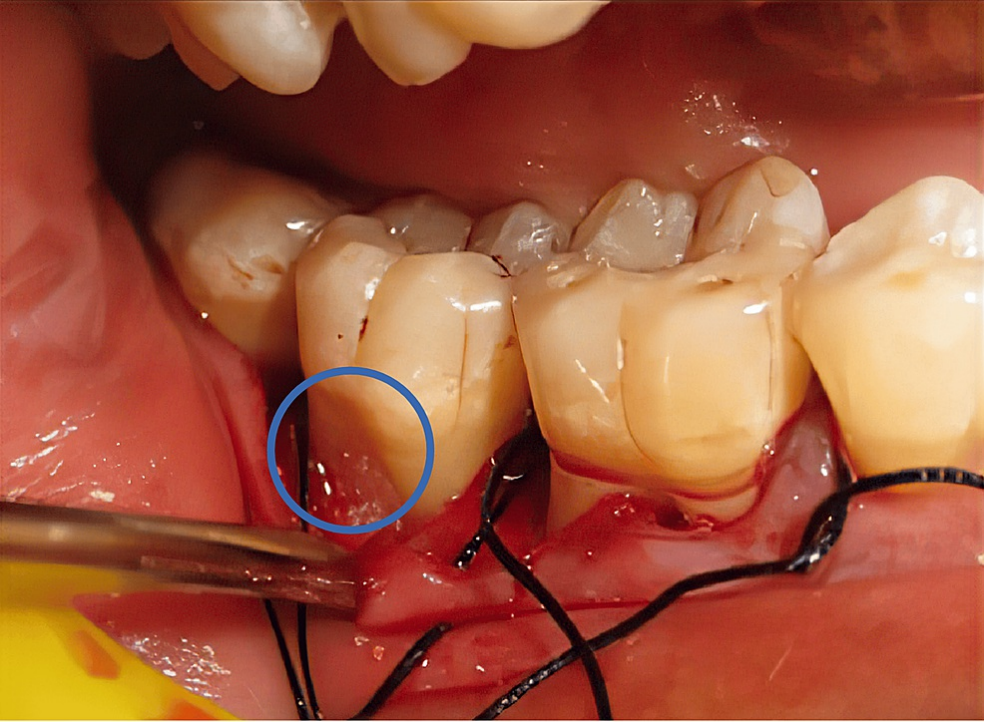
DMBM and PRF placed in the furcation Note: The blue color circle denotes the furcation defect filled with DMBM and autologous PRF fragments

**Figure 3:**
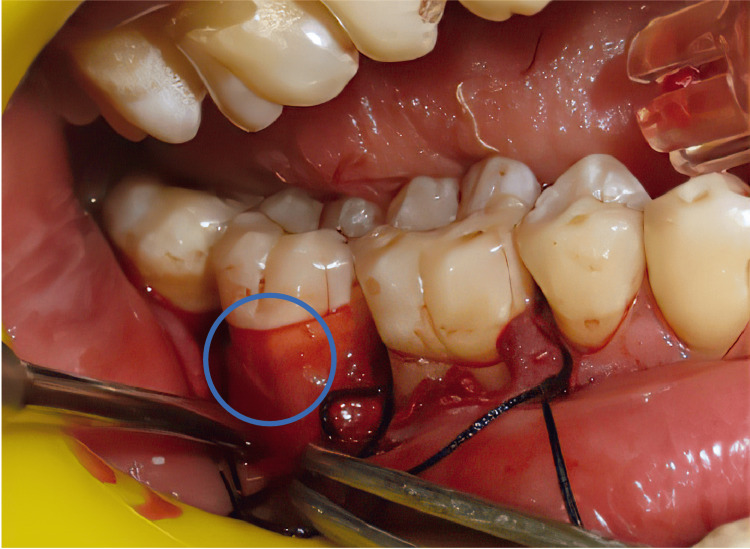
Placement of PRF membrane Note: The blue color denotes the placement of another section of the PRF used as a membrane to cover the furcation

In the test group, the furcation defect was filled with DMBM and autologous PRF fragments (Figure 2), and another section of the PRF was used as a membrane to envelop the furcation. In the control group, components of PRF were inserted into the furcation weak point, and a membrane of PRF was applied to engulf it (Figure 3). The simple interrupted suturing was performed using a medical non-absorbable black silk suture at a thickness of 3-0. The surgical ground was covered and safeguarded with a periodontal sterile covering. Appropriate antimicrobials and analgesics were prescribed to both experimental and control group patients. They were instructed to take 400 mg of ibuprofen three times daily for three days and 500 mg of amoxicillin three times daily for five days. The periodontal dressing and stitches were taken out a week following surgery. Surgical lesions were meticulously cleaned with 0.2% chlorhexidine digluconate. After that, patients were told to brush their teeth slowly with a soft toothbrush. Each patient had a review of good dental hygiene up to one month after surgery and follow-up exams at three and six months.

Ethical approval

This study obtained ethical approval from the Institutional Review Board of the Karnavati School of Dentistry, Karnavati University, Uvarsad-Adalaj Road, Uvarsad, Gandhinagar District, Gujarat-38242, India. Dr. Deepak Shishoo, the Institutional Review Board Chairman, signed the approval certificate dated September 24, 2013. Nonetheless, the ethical approval certificate did not possess any reference number. Therefore, we uploaded the ethical approval certificate as in the Appendices section. Furthermore, all patients and their guardians were informed about the study's objectives, plans, and upcoming scientific publications. Written informed consent and ethical approval certificates were acquired before any interventions were carried out.

Statistical analysis

The univariate regression model by placing the control site as a reference was used to see the mean difference in PD, bone defect depth, and RHCAL between the baseline test and a control site in three and six months. Additionally, regression analysis assessed the extent of an outcome variable concerning an exposure variable. In our study, we calculated the variation in RHCAL between the test and the control site using a β-coefficient for representation. This could also be conveyed as a mean difference. The control site served as the reference point in this comparison. The Mann-Whitney U test was used, while the two sites' mean RVCAL difference was determined. To review the overall changes in PD, bone defect depth, and RHCAL between the test and control site, a repeated measure ANOVA was used to see the statistical difference. A p-value of 0.05 was considered significant. Statistical analysis was performed using STATA 15 (StataCorp LLC, Texas, USA), and the graphical presentation was made by GraphPad Prism 8.3.2 (GraphPad Software, Boston, MA, USA).

## Results

**Figure 4:**
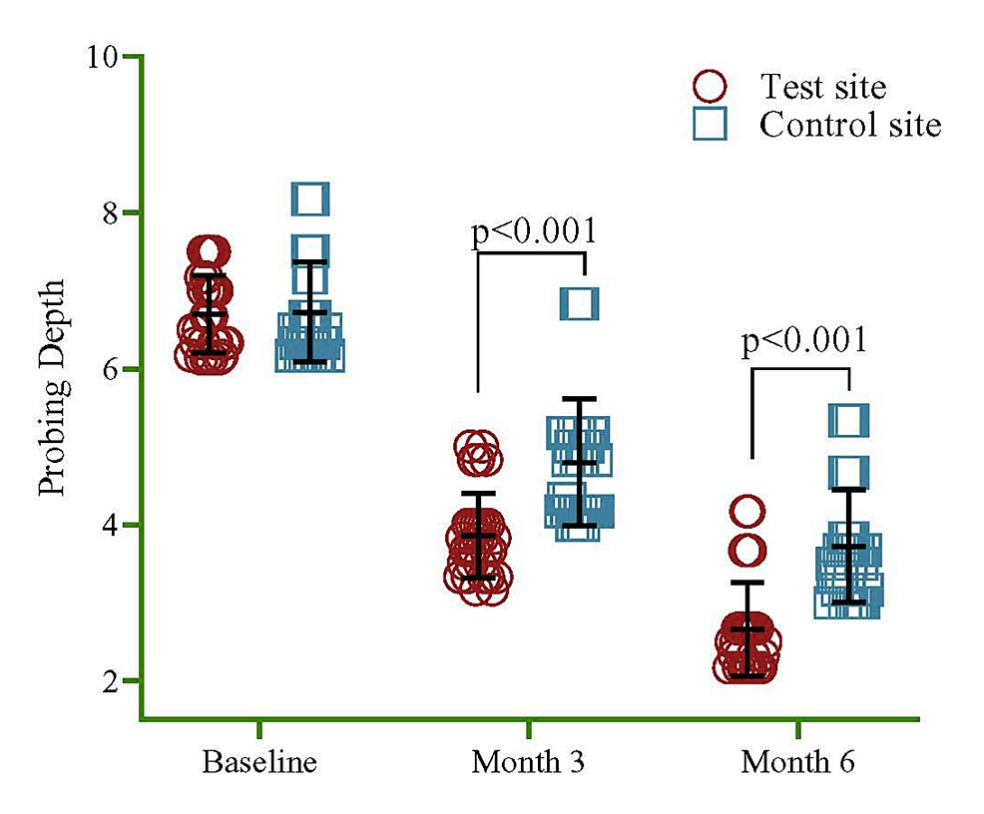
Comparison between the test and control sites in PD at baseline, three months, and six months. The univariate regression model was used to estimate the p-value

At the beginning of the study, the PD was comparable between the test and control sites, with no significant difference observed. However, the univariate regression model showed a significant reduction in PD at the test site compared to the control site (p<0.001), during the three- and six-month follow-up periods. The PD at three months was 3.86±0.54 mm at the test site and 4.80±0.81 mm at the control site, while at six months, it was 2.65±0.60 mm at the test site and 3.72±0.72 mm at the control site (Figure 4). Upon conducting within-group comparisons of PD, the test and control sites exhibited a significant decline from baseline to three and six months. Overall, the PD in the test site was significantly lower than that in the control site, showing a reduction of 68% (95% CI=41%, 95%, p<0.001) (Table 1).

**Table 1:** Overall changes (baseline, three months, and six months) in the test site compared to the control site in PD, bone defect depth, and RHCAL RHCAL: relative horizontal clinical attachment level, PD: probing depth Note: Repeated measured ANOVA was used to estimate the p-value

Test site compared to control site	β-coff (95% CI)	p-value
PD	0.68 (0.41, 0.95)	<0.001
Bone defect depth	0.64 (0.45, 0.83)	<0.001
RHCAL	0.70 (0.31, 0.99)	0.001

**Figure 5:**
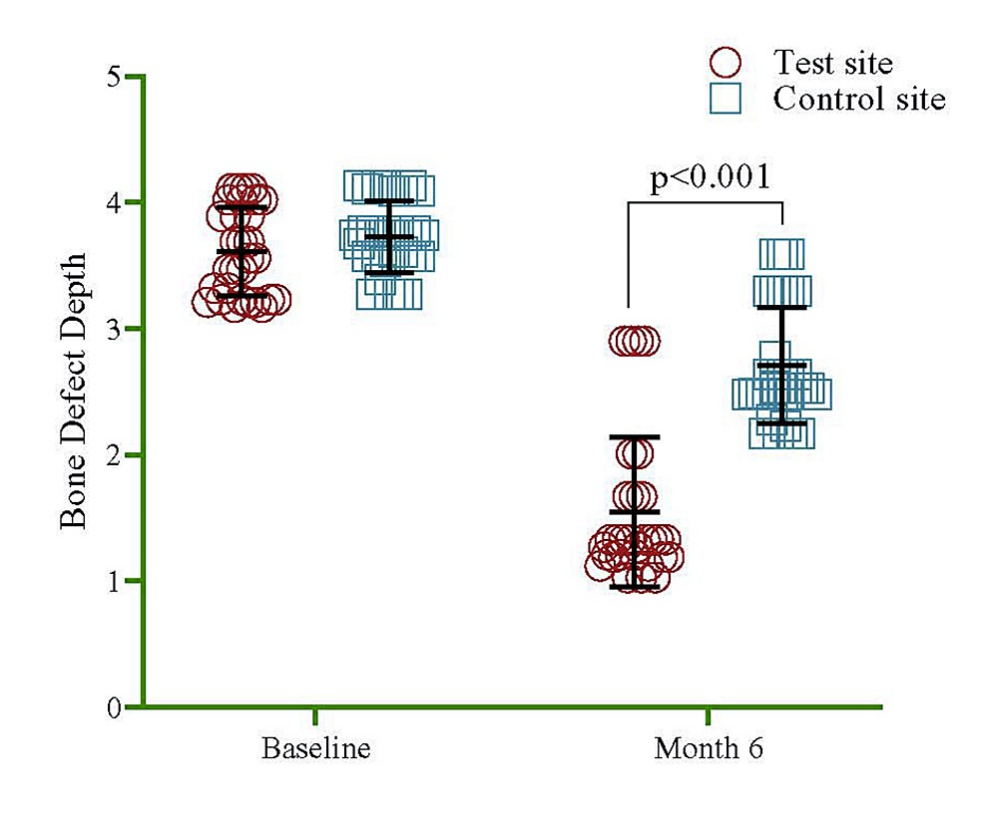
Comparison of the bone defect depth between the test and control sites at baseline and six month. To estimate the p-value, a univariate regression model was utilized

The mean bone defect depth for baseline was 3.61±0.35 in the "test site" group and 3.73±0.28 in the "control" group, respectively. At six months, the mean PD reduced to 1.54±0.59 in the "test site" group and 2.71±0.46 in the "control" group, respectively (Figure 5). Each group had 30 observations at both time points, resulting in a total of 60 observations overall. The output compared the mean values and data spread within and between the groups at different time intervals and showed significance at six months (p<0.001). A significant decline of bone defect depth was noted in the overall period of baseline to six months in the test site by 64% (95% CI=45%, 83%, p<0.001) compared to the control site (Table 1).

**Figure 6:**
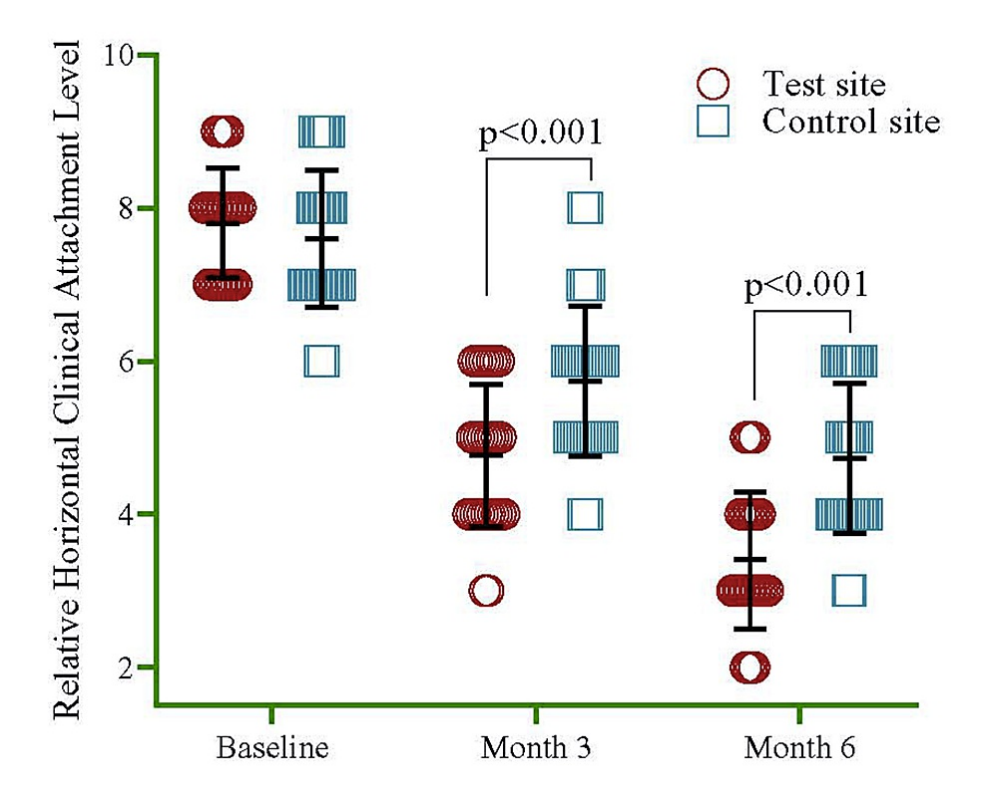
Mean difference of RHCAL between test and control sites at baseline, three months, and six months

At baseline, RHCAL was 7.80±0.71 in the test site and 7.60±0.89 in the control site. By three months, a significant decrease (p<0.001) was observed in the test site (4.77±0.94) compared to the control site (5.73±0.98). Further, at six months, there was another significant reduction (p<0.001) in the test site (3.40±0.89) compared to the control site (4.73±0.98) (Figure 6). The data offers insights into the changes in RHCAL values between the groups over time and the data spread within each group at each time point. Utilizing repeated measure ANOVA for the overall comparison of RHCAL, it was noted that the test site exhibited a significant decline of 70% (95% CI=31%, 99%, p=0.001) compared to the control site (Table 1). When the within-group comparison was made, the test and control sites significantly declined from baseline to three and six months.

**Figure 7:**
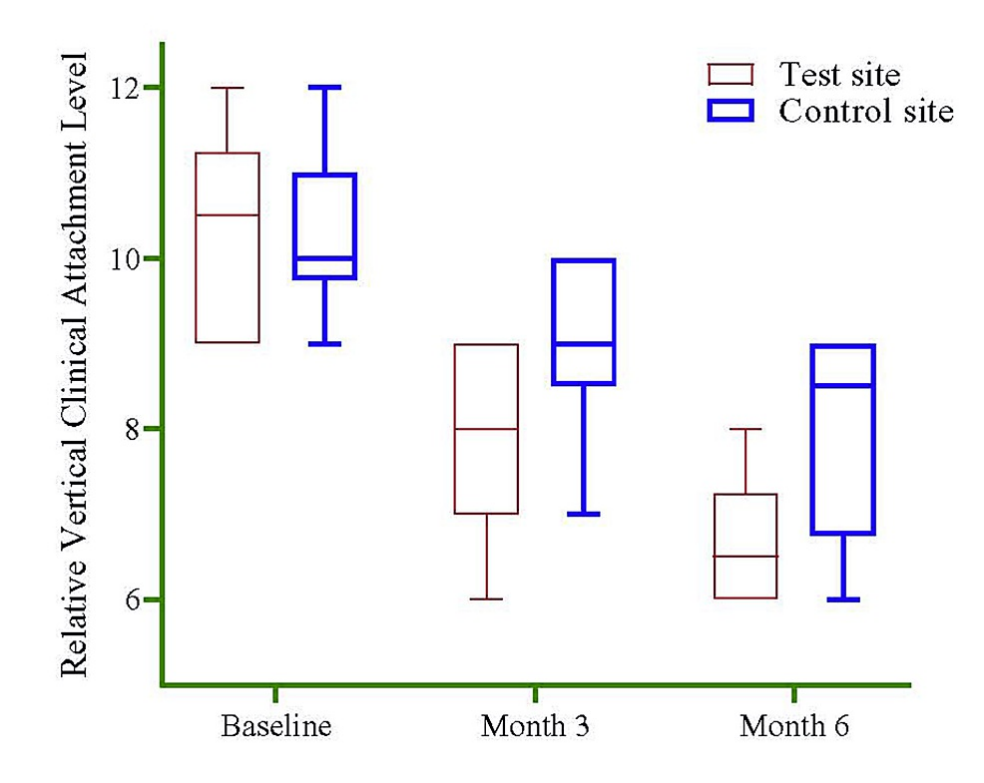
RVCAL difference between the test and control sites Note: The Mann-Whitney U test was used to see the difference between the test and control sites

A sub-group (n=10) observation was collected to see the difference in RVCAL between the test and control sites. Still, no significant difference was noted between the two sites (Figure 7).

**Figure 8:**
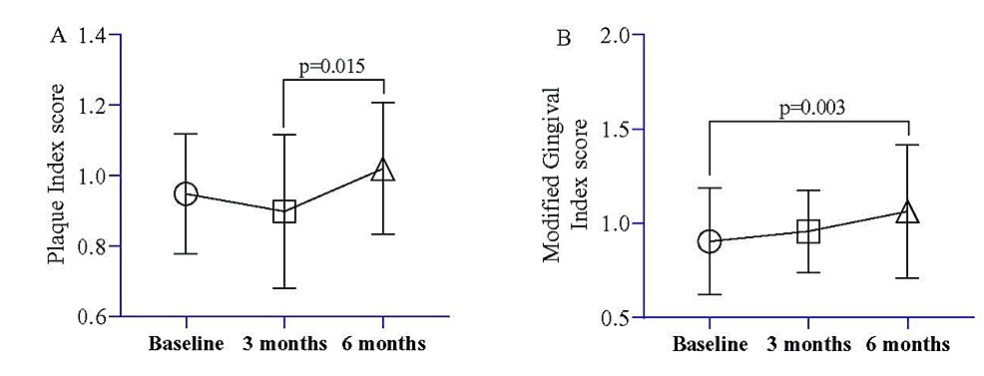
Longitudinal mean comparison of plaque index score (A) and modified gingival index score (B) at baseline, three months, and six months Note: A repeated measure ANOVA was used to estimate the p-value

During the baseline, three months, and six months of assessments, the comparison of plaque and modified gingival indexes was analyzed using repeated measure ANOVA. The results revealed a significant increase in the plaque index between three and six months (p=0.015) (Figure 8A). Additionally, the gingival index score showed a substantial increase at six months compared to baseline (p=0.003) (Figure 8B).

## Discussion

Periodontitis is a complex, persistent inflammatory condition that has an impact on the tissues that support the teeth [52], causing the destruction of periodontal tissues and the development of horizontal and vertical osseous defects [53], which are frequently associated with deep residual pockets, worsening the prognosis for the affected teeth [54,55]. Furcation engrossment results from persistent periodontal disease [56,57]. The current study followed furcation grading constructed by Glickman in 1953 to portray the expansion and foremost features of the furcation imperfection (Grade I-IV) [43,57-59]. The contributing and etiological components must still be mechanically removed [60], and therapeutic interventions such as removing microbes biofilm from the periodontium, supra, and subgingival calculus through professional cleaning, remain the first step of periodontal therapy [61].

The current study aimed to evaluate PRF as an addition to DMBM for treating human periodontal furcation issues. PRP was utilized to fix half of the flaws, while DFDBA and PRP were used to improve the remaining deficiencies. The final assessment at six months was determined using clinical and radiological traits.

Using a blood sample collected without anticoagulants and PRF, one can create a fibrin mesh loaded with platelets and growth factors without artificial biochemical alteration [62-64]. All platelets and growth factors from the blood harvest are concentrated in the PRF clot's natural solid fibrin matrix [65,66]. Additionally, it displays an intricate design that functions as a healing matrix and possesses mechanical qualities that no other platelet concentration can match [67,68]. Multiple studies have revealed that while it promotes the formation of osteoblasts, gingival fibroblasts, and periodontal ligament cells, it suppresses the growth of oral epithelial cells [69-71]. PRF may help heal periodontal osseous abnormalities [72] because it can regulate the expression of phosphorylated extracellular signal-regulated protein kinase [73,74] and prevent osteoclastogenesis by inducing the formation of osteoprotegerin in the bone [73,75,76].

It has been reported that combining a mineralized, rigid bone mineral with a semi-fluid, nonrigid agent, such as EMD, significantly improved the clinical outcome of intra-bony defects compared to treatment without bone materials [74,77]. This is true even though other biological preparations, such as PRP and EMD, are denser and stiffer than PRF [74,78,79]. In a different study, combining PRF and bone minerals improved the effects of regeneration in intra-bony defects [62,74,77,80-82].

**Figure 9:**
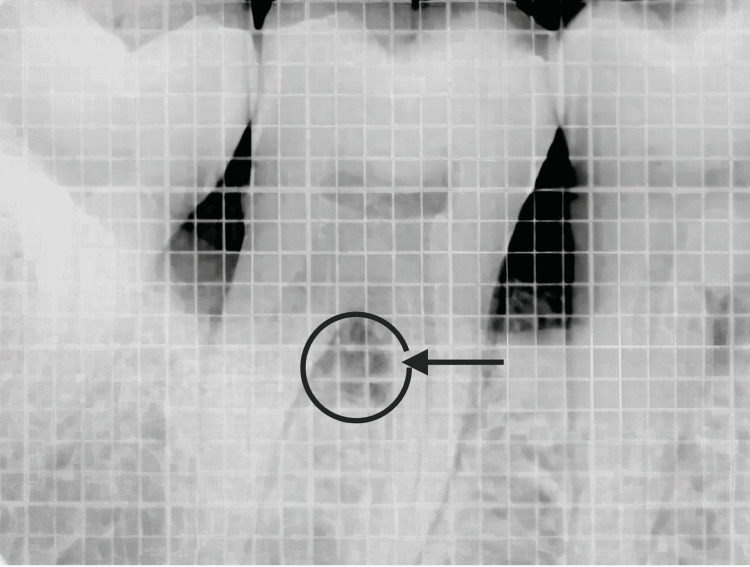
Radiovisiography at baseline showing the furcation defect (circle)

**Figure 10:**
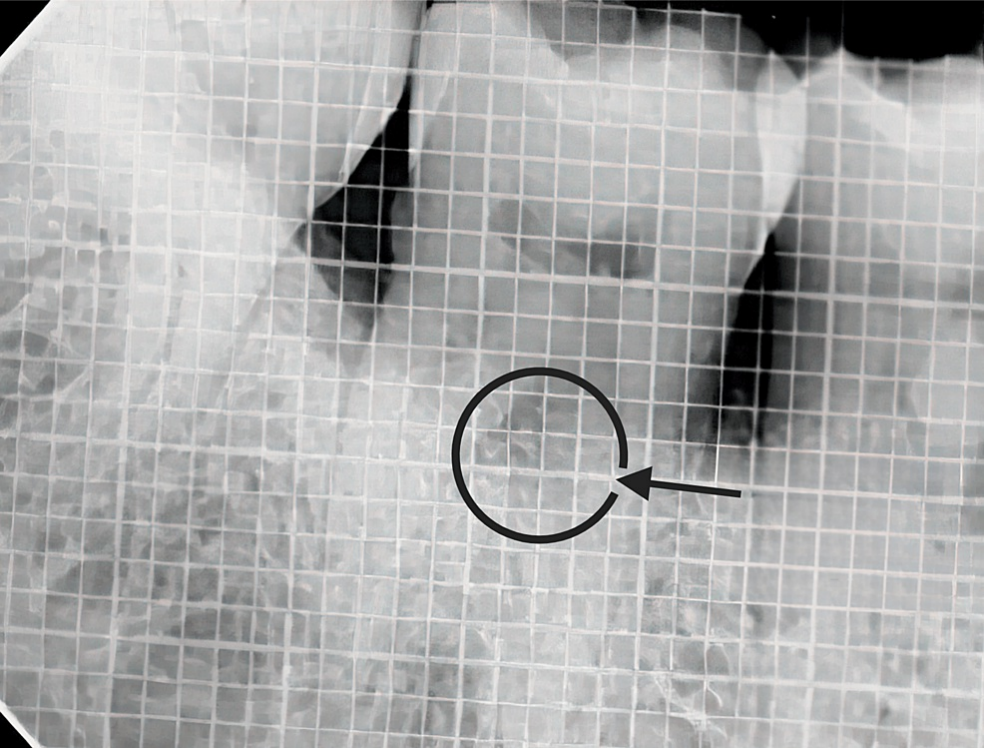
Radiovisiography at six months showing bone fill within the furcation defect (circle)

In a different investigation, PRF and bone minerals exhibited the capacity to enhance the regeneration effects in intrabony defects [74,77,83]. We picked DMBM because we believed it would retain the space necessary for tissue regeneration, improving PRF's efficacy. After six months of follow-up in this clinical research, pocket depth was reduced while clinical attachment was raised. The primary clinical results of all periodontal regeneration therapies are those. Radiographs of the furcation defect revealed significant bone fill compared to baseline readings (Figures 9-10). PRF and xenograft offer a higher level of soft tissue and hard tissue regeneration change in clinical and radiologic parameters when compared to other regenerative materials.

Upon conducting within-group comparisons of PD, the test and control sites exhibited a significant decline from baseline to three and six months. The mean PD at the test site was 6.69 ± 0.50 at baseline, 3.86 ±0.54 at three months, and 2.65 ± 0.60 at six months, indicating a mean difference of 4.04 mm from baseline to six months. Each reading's divergence from the others was statistically significant. At six months, a statistically significant reduction in PD of 3.0 mm was also achieved in the control group; however, more reduction was observed when compared with the test site. Overall, the test site's PD was 68% less than the control site's, a significant difference. Bowers et al. reported that when polytetrafluoroethylene (PTFE) and DFDBA are combined, successful clinical closure of Grade II furcations was achievable at one year [84]. Our study's mean PD reduction at six months was lower than that reported at one year [84]. By implementing a composite graft made of bioabsorbable hydroxyapatite combined with tetracycline hydrochloride and PTFE barrier membrane, Santana et al. observed a significant reduction in PD reduction (3.65 0.6 mm) and more improvements in vertical attachment level and horizontal attachment level [85].

Our study observed that the test and control groups' RVCAL and RHCAL scores significantly improved. In contrast to the intergrade usage of PTFE and DFDBA, which produced a mean gain of more than 1.33 mm in RVCAL, our study presented a mean increase of 3.7 mm in RVCAL.

In the current study, six months after surgery, a mean bone defect depth reduction of 4.04 mm in the test sites and 3.0 mm in the control sites was noted. As a result, the test sites showed a statistically significant cutting down of the depth of bony defect compared to the control sites. Concerning these factors, we found that the mean bone fills in the test group was 57.39% and 28.64% in the control group, which clearly shows superior healing at the test location after six months. Multiple studies also supported this outcome [86-88]. They tested how PRP and Bio-Oss affected bone reclamation and renewal in animal subjects with Grade II furcation defects. Results indicated that when PRP and Bio-Oss were combined to treat Grade II furcation deficiencies, 61% of the bone was filled [86-88]. At nine months after surgery, open flap debridement alone is less effective for treating horizontal periodontal abnormalities than PRF, either in gel or membrane form. In a study by Bansal et al., surgical intervention regarding periodontal intrabony disorders with DFDBA and autologous PRF had better clinical end results [89].

**Figure 11:**
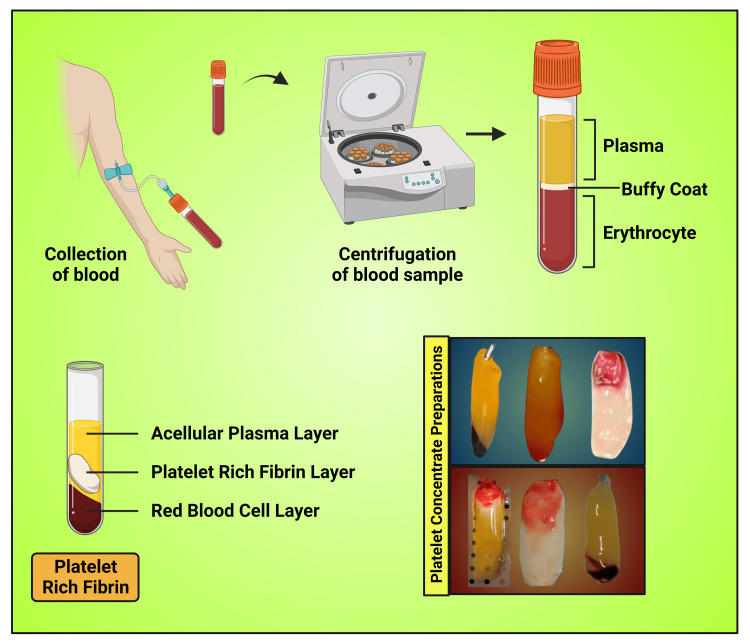
Schematic diagram showing clinical preparation of PRF Note: This image was created using the premium edition of BioRender (https://biorender.com/accessed on August 18, 2023) with the license number AY25QU0LD0. Image credit: Susmita Sinha

According to recent studies by Zhou et al. that involved a meta-analysis of the adjunctive effects of bioactive materials like PRP, PRF, EMD, and amnion membrane, as well as bone grafting for periodontal intrabony abnormalities, PRF and PRP dramatically improved PD reduction and CAL gain. Only PRF showed a successful reduction in the recession [24]. Atchuta et al. reported that the DFDBA and PRF groups had the highest decreases in PPD and radiographic defect depth [82]. DFDBA encompasses bone morphogenetic protein (BMP) that instigates fresh bone construction during the bone restorative phase and is wished for as an alternative for autologous bone in dental and oral surgical procedures [90-92]. DMBM is a type of bone graft with osteoconductive and osteoinductive marketable biomaterial and permitted medical device cast-off in bone blemishes with a prolonged history of clinical use in miscellaneous medical procedures. It is managed from a human allograft bone [93,94]. In a systematic review and meta-analysis by Tarallo et al., PRF was used to treat Grade II furcation defects. It demonstrated superior results than open flap debridement alone in furcation treatment [95] (Figure 11). According to a 2017 study, PRF promotes excellent soft tissue repair compared to rhBMP-2. Furthermore, it is impossible to disregard the additional advantages of PRF's accessibility and cost [9,96].

**Figure 12:**
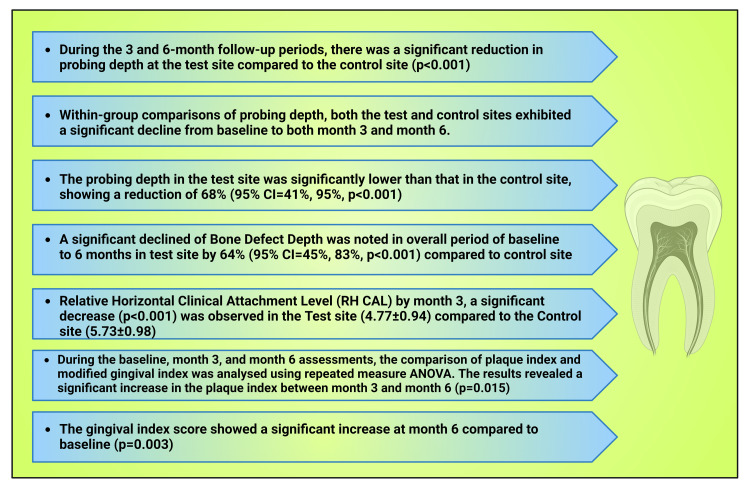
Chart showing the study findings Notes: This figure has been drawn with the premium version of BioRender (https://biorender.com/accessed on August 18, 2023) with the license number XW25QQULXN. Image credit: Susmita Sinha

Despite having positive clinical outcomes, histopathological analysis was not performed in the current investigation. Therefore, it is unable to provide definitive proof of periodontal regeneration. Additionally, our study's sample size was not particularly big. Thus, further research with more participants and histologic examination may shed more light on the advantages of combining PRF and DMBM (Figure 12). A longer postoperative therapy observation interval may be required to validate the stability of clinical outcomes.

Limitations of the study

A limitation of this study is that we did not collect baseline characteristics of the participants. Consequently, we were not able to adjust for any covariates to mitigate the potential influence of confounding factors. This lack of baseline data might impact the comprehensive understanding of participant characteristics at the study's outset.

## Conclusions

Therefore, it can be said that PRF and DMBM together significantly improved clinical indicators compared to PRF alone. In the test group, more bone fill was seen on radiographs. Furthermore, the univariate regression model showed a significant reduction in PD at the test site compared to the control site (p<0.001) during the three- and six-month follow-up periods. In addition to promoting wound healing by providing growth factors, graft stabilization, homeostasis, and increasing the handling capabilities of the graft materials are also benefits of using PRF in the gel form in conjunction with bone grafts.

## Figures and Tables

**Figure 13:**
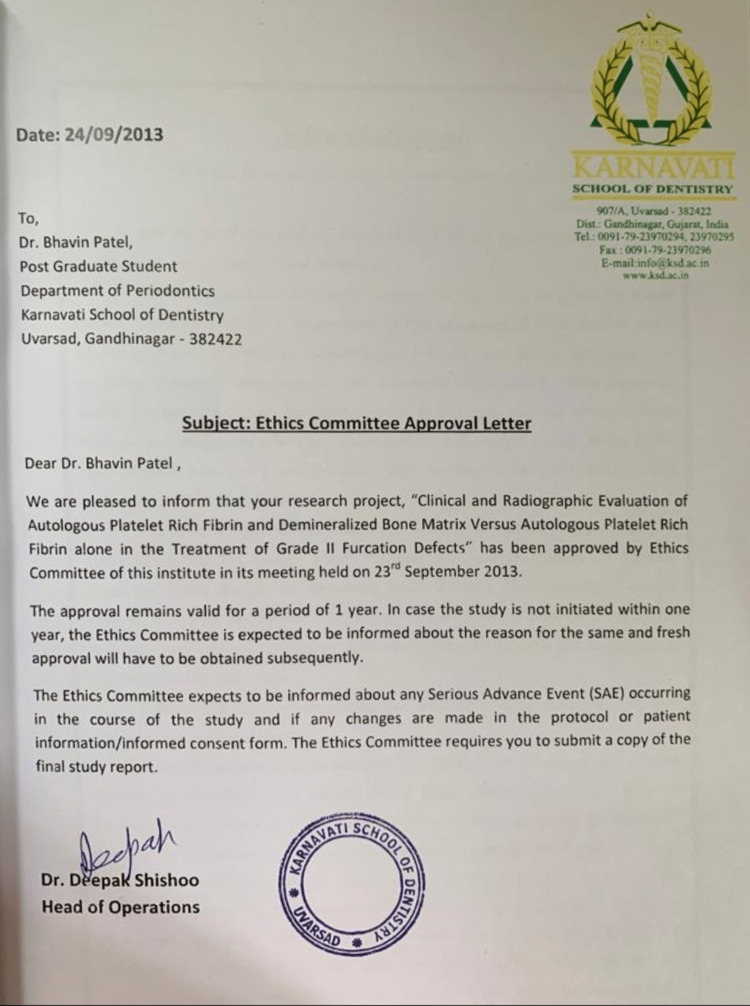
Certificate of ethical approval
